# The relationship between ultra processed food consumption and premature coronary artery disease: Iran premature coronary artery disease study (IPAD)

**DOI:** 10.3389/fnut.2023.1145762

**Published:** 2023-06-30

**Authors:** Shakila Ansari, Noushin Mohammadifard, Fahimeh Haghighatdoost, Ehsan Zarepur, Shirin Mahmoudi, Fatemeh Nouri, Fereidoon Nouhi, Hassan Alikhasi, Fariborz Sharifianjazi, Ketevan Tavamaishvili, Shahin Shirani, Tooba Kazemi, Nahid Azdaki, Nahid Salehi, Masoud Lotfizadeh, Kamal Solati, Samad Ghaffari, Elmira Javanmardi, Arsalan Salari, Mostafa Dehghani, Mostafa Cheraghi, Ahmadreza Assareh, Habib Haybar, Seyedeh M. Namayandeh, Reza Madadi, Nizal Sarrafzadegan

**Affiliations:** ^1^Department of Community Nutrition, Nutrition and Food Security Research Center, School of Nutrition and Food Sciences, Isfahan University of Medical Sciences, Isfahan, Iran; ^2^Interventional Cardiology Research Center, Cardiovascular Research Institute, Isfahan University of Medical Sciences, Isfahan, Iran; ^3^Isfahan Cardiovascular Research Center, Cardiovascular Research Institute, Isfahan University of Medical Sciences, Isfahan, Iran; ^4^Department of Cardiology, Medicine School, Isfahan University of Medical Sciences, Isfahan, Iran; ^5^Rajaie Cardiovascular Medical and Research Center, Iran University of Medical Sciences, Tehran, Iran; ^6^Iranian Network of Cardiovascular Research (INCVR), Tehran, Iran; ^7^Heart Failure Research Center, Cardiovascular Research Institute, Isfahan University of Medical Sciences, Isfahan, Iran; ^8^School of Science and Technology, The University of Georgia, Tbilisi, Georgia; ^9^Georgian American University, Tbilisi, Georgia; ^10^Department of Cardiology, Tehran University of Medical Science, Dr Ali Shariati Hospital, Tehran, Iran; ^11^Cardiovascular Diseases Research Center, Birjand University of Medical Sciences, Birjand, Iran; ^12^Clinical Research Development Unit, Razi Hospital, Birjand University of Medical Sciences, Birjand, Iran; ^13^Cardiovascular Research Center, Health Institute, Kermanshah University of Medical Sciences, Kermanshah, Iran; ^14^Social Determinants of Health Research Center, Shahrekord University of Medical Sciences, Shahrekord, Iran; ^15^Department of Psychiatry, Shahrekord University of Medical Science, Shahrekord, Iran; ^16^Cardiovascular Research Center, Tabriz University of Medical Sciences, Tabriz, Iran; ^17^Department of Cardiovascular Medicine, Amiralmomenin Hospital, Maragheh University of Medical Sciences, Maragheh, Iran; ^18^Cardiovascular Diseases Research Center, Department of Cardiology, Heshmat Hospital, School of Medicine, Guilan University of Medical Sciences, Rasht, Iran; ^19^Department of Cardiovascular Research Center, Shahid Rahimi Hospital, Lorestan University of Medical Science, Khorramabad, Iran; ^20^Atherosclerosis Research Center, Ahvaz Jundishapur University of Medical Sciences, Ahvaz, Iran; ^21^Yazd Cardiovascular Research Center, Shahid Sadoughi University of Medical Science, Yazd, Iran; ^22^Center for Healthcare Data Modeling, Departments of Biostatistics and Epidemiology, School of Public Health, Shahid Sadoughi University of Medical Sciences, Yazd, Iran; ^23^Zanjan University of Medical Sciences, Zanjan, Iran; ^24^Faculty of Medicine, School of Population and Public Health, University of British Columbia, Vancouver, BC, Canada

**Keywords:** ultra-processed food, premature coronary artery disease, coronary artery diseases, cardiovascular disease, processed food

## Abstract

**Background:**

Ultra-processed foods (UPF) consumption may affect the risk of PCAD through affecting cardio metabolic risk factors. This study aimed to evaluate the association between UPFs consumption and premature coronary artery disease (PCAD).

**Methods:**

A case–control study was conducted on 2,354 Iranian adults (≥ 19 years). Dietary intake was assessed using a validated 110-item food frequency questionnaire (FFQ) and foods were classified based on the NOVA system, which groups all foods according to the nature, extent and purposes of the industrial processes they undergo. PCAD was defined as having an stenosis of at least single coronary artery equal and above 75% or left main coronary of equal or more than 50% in women less than 70 and men less than 60 years, determined by angiography. The odds of PCAD across the tertiles of UPFs consumption were assessed by binary logistic regression.

**Results:**

After adjustment for potential confounders, participants in the top tertile of UPFs were twice as likely to have PCAD compared with those in the bottom tertile (OR: 2.52; 95% CI: 1.97–3.23). Moreover, those in the highest tertile of the UPFs consumption had more than two times higher risk for having severe PCAD than those in the first tertile (OR: 2.64; 95% CI: 2.16–3.22). In addition, there was a significant upward trend in PCAD risk and PCAD severity as tertiles increased (P-trend < 0.001 for all models).

**Conclusion:**

Higher consumption of UPFs was related to increased risk of PCAD and higher chance of having severe PCAD in Iranian adults. Although, future cohort studies are needed to confirm the results of this study, these findings indicated the necessity of reducing UPFs intake.

## Introduction

Coronary artery disease (CAD) is the most frequent cardiac complication of chronic diseases in developing countries (1). In general, CAD occurs in old ages, but some studies have reported that almost 4–10% of the patients are young (2). Premature coronary artery disease (PCAD) affects women younger than 45 and men younger than 55 years, but these cut-offs vary from 45 to 70 years in different studies (3–5). CAD causes around 350,000 deaths per year globally (6) and accounts for approximately 50% of all deaths each year in Iran (7). A healthy eating pattern may reduce the risk of heart disease by 50% (8), which is mediated by the effect of dietary habits on multiple risk factors of cardiovascular disease (CVD). Ultra-processed foods (UPF) consumption is one of the important factors which considerably affect diet quality and nutrients intake (9) and influence the risk of various cardio metabolic risk factors (10, 11).

There are various food classifications based on the level of processing amongst them the NOVA system is the most common (12). Accordingly, UPFs group refers to industrial manufactured foods like, breakfast cereals, biscuits, fast foods, sodas, ice-cream, and frozen meals (13). Most of the UPFs contain modified starch, sodium, hydrogenated oils, colors and classes of additives which all lead to production of compounds with potential cardio-metabolic effects (14). Phosphate which is widely used in UPFs in order to enhance appearance, and shelf life, can lead to atherosclerosis by promoting vascular calcification (15). While UPFs are high dense calorie products, they are poor in nutrients (9). Providing a major daily energy intake from UPFs deprives the intake of fresh and less processed foods, which indirectly endangers health (16). Finally, UPFs underlie obesity (11), hypertension, dyslipidemia and hyperglycemia (17) which all are risk factors for CVD (18). The NutriNet-Santé cohort study on French adult found that every 10% increment in UPFs consumption was linked with 12% increase in the risk of CVD (19). Consistently, the Moli-sani study on a large sample of the Italian adult population revealed that higher amount of dietary UPFs increased the risk of CVD and all-cause mortality (20). In contrast, a Brazilian study found that the higher UPFs intake was in line with the decreased odds of the simultaneous presence of CAD, peripheral arterial diseases, and stroke (21).

Due to the fact that UPFs have a long shelf life, are tasty, ready to use, and most importantly are not expensive (22) the tendency toward UPFs consumption is increasing worldwide (23). The individual consumption data revealed that UPFs consumption accounted for 60% and up to 36% of daily energy intake in the United State and European countries, respectively, (20). In contrast, due to the low consumption of UPFs reported in previous studies in Iran [8–20%; (9, 11) of daily energy intake], it is possible to obtain different relations compared to previous studies.

Although there are several studies on the relationship between UPFs and CVD, so far, no study has been done specifically on PCAD. Also, due to differences among lifestyle, dietary habits and the average consumption of UPFs in each population, there may be contradictions between the results of different studies. Therefore, this study aims to investigate the association between UPFs consumption and the PCAD in a multi-centric study of Iranians.

## Materials and methods

### Participants

This case–control study was conducted using the data from IRAN Premature Coronary Artery Disease study (IPAD) (24). IPAD is a multi-center case-control study with the primary objective of determining the frequency of PCAD and its related risk factors in different ethnicities of Iranians including Fars, Azari, Kurd, Arab, Lor, Gilak, Balouch, Turkman, Qashqaei, and Bakhtiari. The inclusion criteria were females less than 70 and males less than 60 years who underwent coronary an giography and had an stenosis of at least single coronary artery equal and above 75% or left main coronary of equal or more than 50% in the cases and normal coronary artery in control group. Previous history of documented CAD, such as coronary artery bypass surgery, balloon angioplasty, or percutaneous coronary intervention was considered exclusion criteria. A total of 2,351 eligible patients were selected *via* convenient sampling from reference hospitals in 16 cities of Iran. This study was reviewed and approved by the Ethics Committee of Isfahan University of Medical Sciences (IR.MUI.REC.1396.2.055). We obtained a written informed consent from all patients and the Declaration of Helsinki was considered.

### Data collection

Trained healthcare professionals completed questionnaires and collected data regarding demographics characteristics, personal and family history of illnesses and medications. A demographic questionnaire was used to collect marital status and level of education. The height of patients was measured by a wall-fixed stadiometer with a sensitivity of 0.1 cm while participants were barefoot, and weight was obtained while participants were minimally clothing and recorded to the nearest 0.1 kg. Body mass index (BMI) was calculated by dividing weight in kilogram by height squared in meter. Waist circumference (WC) was measured with a tape measure between the lowest rib and iliac crest to the nearest 0.1 cm (25). Systolic and diastolic blood pressures (SBP and DBP) were measured by a digital sphygmomanometer (BC 08, Beurer, Germany) after being seated for at least 5 min. Blood pressure was measured twice per person and the average value was recorded.

### Dietary assessment

Data regarding usual dietary intakes of participants over the past year were obtained *via* a validated and reliable 110-item semi-quantitative food frequency questionnaire (FFQ) (26). The FFQ provided the frequency in ten-option categories (‘seldom/never, once per month, 2–3 per month, once per week, 2–3 per week, 4–6 per week, 1 per day, 2–3 per day, 4–5 per day, and 6 or more per day). In addition, they were asked to record the portion sizes of each food and beverage items. Using household measurements, the frequency was converted to daily intake and multiplied by the portion sizes were changed to gram. Portion sizes of foods and beverages were also obtained. Subsequently, the daily intake of each nutrient, UPFs and daily energy intake were determined by using Iranian Food Consumption Processor and food composition table (RE) (27, 28).

Ultra-processed foods were determined according to the NOVA classification system (8). NOVA classifies foods into four groups based on the amount of processing done on the foods. The first group consists of unprocessed or minimally processed foods like vegetables and fruits, the second group refers to processed culinary ingredients, like sugar, and salt, the third group includes processed foods, like canned foods, and cheeses, and the last group are UPFs which are industrial compounds without or with the least amount of whole foods and with notable amounts of colors, flavors and preservatives. Accordingly, foods with specific formulations of ingredients which are produced by a series of industrial processes were regarded as ultra-processed foods in our study These foods include pre-prepared dishes, industrial packaged breads, breakfast cereals, confectionery, biscuits, pastries, buns, and cakes, fast food dishes, reconstituted meat products, sweetened milk-based products chips, crackers, and other salty snacks, margarine and other spreads, sauces, dressing and gravies, industrial desserts, soft drinks, sweetened fruit juices, and drinks (29). Pre-prepared pasta, stuffed pasta, frozen (mixed) dishes, and alcoholic beverages (wine, beer, spirits) were not included in the UPFs list of this study since their consumption is not common in the Iranian diet and do not substantially contribute to daily energy and nutrient intake.

### Biochemical assessment

Blood samples were collected after a fasting period of 12 h to measure fasting blood sugar (FBS), triglyceride (TG), total cholesterol (TC), high-density lipoprotein cholesterol (HDL-C), and low-density lipoprotein cholesterol (LDL-C). Serum lipids and glucose were measured using the enzymatic method, based on calorimetry, utilizing commercial kits (Pars Azmoun, Iran) with an automatic device (Selecta E, Vitalab, Netherlands). Based on the definition of the National Cholesterol Education Program (NCEP) TG ≥ 150 mg/dl, TC ≥ 240 mg/dl, LDL ≥ 160 mg/dl, and HDL-C < 40 in men and < 50 mg/dl in women, were considered as hypertriglyceridemia, hypercholesterolemia, raised LDL-C and low HDL-C, respectively (30).

### Statistical analysis

All analyses were performed using SPSS Statistics 26.0 software (IBM, Armonk, NY, United States). Two-tailed *p*-values were reported and statistical significance was considered *p* < 0.05. Kolmogorov Smirnov test was used to explore the normality of the distribution for quantitative variables. The tertiles of UPFs were determined based on the overall range of UPFs consumption in the whole population. General characteristics of participants across the tertiles of UPFs were compared by one-way analysis of variance (ANOVA) for continuous variables such as BMI, age and WC and Chi-square test for categorical variables. The independent-sample Student *t*-test was used to distinguish means of continuous variables between cases and controls. For comparing the distribution of participants in categorical variable levels between cases and controls, the chi-square test was run. The association between UPFs consumption and PCAD was assessed by binary logistic regression in different models. The crude model remained unadjusted. Model 1 was adjusted for age, sex, and daily energy intake. Model 2 was additionally adjusted for smoking, level of education, different ethnicities and family history of CVD. Model 3 was additionally adjusted for the Mediterranean dietary score. BMI in model 4, and WC in model 5 were additionally and separately adjusted as mediators. Due to the small sample size of some subgroups of ethnicities, the association between UPFs consumption and PCAD in different ethnicities was assessed by binary logistic regression in just one adjusted model for age, sex, and daily energy intake.

## Results

General characteristics of cases and controls across the tertiles of UPFs consumption are presented in Table 1. On average, the contribution of UPFs to daily energy intake was 6.55% kcal/day (SD: 5.71). Ceases were older, more likely to have family history of CVD (*p*-value < 0·001), hypertriglyceridemia (*p*-value < 0·001) and hypertension (*p*-value: 0.308). The average of FBS and SBP levels were significantly higher in cases than that of in controls (*p*-value < 0·001 and 0.044 respectively). In comparison to controls, cases showed lower LDL-C, HDL-C and TC mean values (*p*-value < 0·001). Assessing participants’ characteristics across the tertiles of UPFs consumption revealed that individuals in the top tertile of UPFs consumption were younger (*p*-value: < 0·001), more educated (*p*-value: < 0·001) more frequently current smokers (*p*-value: < 0·001) and had lower TC levels (*p*-value: 0.033).

**Table 1:** General characteristics of participants based on case and control and across tertiles of ultra-processed foods consumption.

	Participants group		*p* Value^1^	UPFs consumption tertiles (% of Kcal)			*p* Value^2^
	Case	Control		T1 (mean ± S*D* = 1.9 ± 1.0)	T2 (mean ± SD = 5.5 ± 1.2)	T3 (mean ± SD = 13.7 ± 6.0)	
Age, years	54.7 ± 7.2	51.9 ± 8.3	<0.001	55.2 ± 7.2	53.8 ± 7.7	52.1 ± 8.0	<0.001
Ethnicity *n* (%)							
Fars	1,096 (52.9%)	655 (56.4%)	<0.001	539 (50.1%)	613 (56.8%)	639 (59%)	0.002
Azari	213 (10.3%)	82 (7.1%)		95 (8.8%)	83 (7.7%)	72 (6.6%)	
Kurd	193 (9.3%)	174 (15%)		138 (12.8%)	114 (10.6%)	112 (10.3%)	
Lor	126 (6.1%)	23 (2%)		65 (6.0%)	50 (4.6%)	48 (4.4%)	
Bakhtiari	136 (6.6%)	73 (6.3%)		85 (7.9%)	67 (6.2%)	55 (5.1%)	
Qashqaei	76 (3.7%)	56 (4.8%)		49 (4.6%)	46 (4.3%)	36 (3.3%)	
Arab	64 (3.1%)	31 (2.7%)		21 (2.0%)	31 (2.9%)	43 (4.0%)	
Gilak	167 (8.1%)	65 (5.6%)		83 (7.7%)	74 (6.9%)	75 (6.9%)	
Balouch	2 (0.1%)	3 (0.3%)		1 (0.1%)	1 (0.1%)	3 (0.3%)	
Sex Men n (%)	1,358 (65.4%)	423 (36.1%)	<0.001	495 (45.8%)	598 (55.1%)	696 (64%)	<0.001
BMI, Kg/m^2^	*27.9 ± 4.5*	28.8 *± 5.1*	<0.001	28.2 *± 4.7*	28.3 *± 4.8*	28.2 *± 4.7*	0.806
Marital status Married, *n* (%)	*1884 (90.7%)*	1,033 (88.1%)	0.023	962 (89.1%)	983 (90.5%)	980 (90.1%)	0.522
Education level, *n* (%)							
*Undergraduate*	*1,022 (49.2%)*	663 (56.6%)	<0.001	682 (63.2%)	543 (50%)	439 (40.3%)	<0.001
*Graduate*	*802 (38.6%)*	414 (35.4%)		320 (29.7%)	423 (39%)	489 (44.9%)	
*Postgraduate*	*253 (12.2%)*	94 (8%)		77 (7.1%)	120 (11%)	160 (14.7%)	
Current smoking, *n* (%)	*514 (24.7%)*	159 (13.6%)	<0.001	150 (13.9%)	230 (21.2%)	288 (26.5%)	<0.001
Family history of CVD (%)	*1,095 (54.7%)*	517 (45.1%)	<0.001	510 (48.6%)	531 (50.5%)	580 (54.7%)	0.015
Type 2 diabetes, *n* (%)	*701 (98.5%)*	265 (97.4%)	0.282	356 (98.1%)	296 (99%)	304 (97.7%)	0.474
High WC, cm	*99.6 ± 11.6*	98.5 *± 12.4*	0.010	98.7 *± 11.5*	99.7 *± 11.8*	99.1 *± 12.1*	0.150
Hypertension, *n* (%)	*851 (93.9%)*	443 (95.3%)	0.308	459 (95.2%)	443 (94.9%)	393 (92.9%)	0.274
Dyslipidemia, *n* (%)	*1,493 (78.1%)*	844 (76.4%)	0.288	800 (79.1%)	784 (77.6%)	728 (76.7%)	0.427
Hypertriglyceridemia, *n* (%)	*834 (43.8%)*	414 (37.6%)	0.001	419 (41.6%)	404 (40.1%)	415 (43.9%)	0.240
FBS, mg/dL	*121.2 ± 55.9*	106.1 *± 39.7*	<0.001	116.1 *± 51.3*	113.7 *± 48.9*	116.5 *± 52.6*	0.446
SBP, mmHg	*122.5 ± 19.1*	121.2 *± 16.7*	0.044	122.9 *± 18.2*	122.6 *± 18.6*	120.8 *± 18.0*	0.014
DBP, mmHg	*78.1 ± 11.6*	77.6 *± 10.6*	0.202	77.8 *± 11.3*	78.4 *± 11.4*	77.7 *± 11.3*	0.331
TC, mg/dL	*158.4 ± 44.4*	175.5 *± 42.8*	<0.001	167.1 *± 47.3*	164.9 *± 43.5*	161.8 *± 43.3*	0.033
HDL-C, mg/dL	*41.8 ± 10.9*	45.9 *± 11.5*	<0.001	43.4 *± 11.7*	43.3 *± 11.1*	43.0 *± 10.9*	0.674
LDL, mg/dL	*86.5 ± 33.6*	99.9 *± 32.5*	<0.001	92.9 *± 37.1*	91.2 *± 32.1*	90.1 *± 32.3*	0.180
TG, mg/dL	*158.1 ± 85.9*	147.7 *± 78.1*	0.001	155.9 *± 84.2*	153.0 *± 79.7*	155.6 *± 84.6*	0.684
Physical activity	*1990.1 ± 2358.4*	1812.1 *± 2505.9*	0.063	2042.6 *± 2553.8*	1862.4 *± 2435.1*	1860.8 *± 2168.2*	0.177

1 Independent *t*-test was used for continues variables, and Chi-square test for categorical variables.

2 Analysis of variance was used for continues variables, and Chi-square test for categorical variables.

Data are mean ± SD, otherwise indicated.

UPF: ultra processed food; SD: Standard deviation; BMI: body mass index; WC: waist circumference; FBS: fasting blood sugar; SBP: systolic blood pressure; DBP: diastolic blood pressure; TC: total cholesterol; HDL-C: high density lipoprotein cholesterol; LDL-C: low density lipoprotein cholesterol; TG: triglyceride.

Dietary intakes of cases and controls across the tertiles of UPFs consumption are indicated in Table 2. Cases had higher intake of saturated fatty acids (SFAs; *p*-value: < 0·001), cholesterol (*p*-value: < 0·001), dairy products (*p*-value: < 0·001), cake and cookies, soft drinks, sweets, processed meats and mayonnaise and dressings (*p*-value: < 0·001) than that of in control group. Total fat (*p*-value: < 0·001), protein (*p*-value: < 0·001), Fruits and Vegetables (*p*-value: < 0·001), monounsaturated fatty acids (MUFAs; *p*-value: 0.024) and polyunsaturated fatty acids (PUFAs; *p*-value: < 0·001) dietary intakes of controls were significantly higher than cases. Evaluating dietary intakes of participants across the tertiles of UPFs consumption showed that individuals in the highest tertile of UPFs consumption had higher intake of total energy, SFAs, PUFAs (*p*-value: 0.012), cholesterol, cake and cookies, soft drinks, sweets, processed meats and mayonnaise and dressings (*p* for all < 0·001) than participants in the first tertile. Bread, sodium (sodium content of table salt and cooking salt were not measured), protein and fiber intake of those in the third tertile of UPFs consumption was significantly lower than whom in the first tertile (*p*-value: < 0·001 for all).

**Table 2:** Dietary intakes of participants based on case and control and across tertiles of ultra-processed food (UPFs) consumption.

Dietary intake	Participants group		*p* Value^1^	UPF consumption tertiles (% of Kcal)			*p* Value^1^
	Case	Control		T1	T2	T3	
Energy intake (Kcal/day)	*2047.6* ± 18.1	2096.2 ± 24.5	0.125	1959.2 ± 24.3	2038.3 ± 24.0	2225.1 ± 24.2	<0.001
Nutrients (g/day)							
Proteins	*82.3* ± 0.4	84.8 ± 0.5	<0.001	86.6 ± 0.5	83.8 ± 0.5	79.7 ± 0.5	<0.001
Total fat	*83.9* ± 0.5	88.8 ± 0.7	<0.001	85.1 ± 0.7	87.5 ± 0.7	86.1 ± 0.7	0.055
Saturated fatty acids	*32.7* ± 0.3	*30.7* ± 0.4	<0.001	*31.3* ± 0.4	*32.1* ± 0.4	*33.4* ± 0.4	<0.001
Monounsaturated fatty acids	*25.4* ± 0.2	26.2 ± 0.3	0.024	25.6 ± 0.3	26.1 ± 0.3	26.0 ± 0.3	0.321
Polyunsaturated fatty acids	*21.4* ± 0.2	25.7 ± 0.3	<0.001	22.7 ± 0.3	23.8 ± 0.3	23.0 ± 0.3	0.012
Cholesterol	*293.6* ± 3.0	260.2 ± 4.0	<0.001	268.5 ± 4.0	281.9 ± 3.9	300.6 ± 4.0	<0.001
Fibers	21.4 ± 0.1	21.8 ± 0.2	0.068	22.7 ± 0.2	21.5 ± 0.2	20.6 ± 0.2	<0.001
Sodium	*2090.1* ± 16.2	2067.4 ± 21.9	0.422	2215.1 ± 21.6	2051.7 ± 21.2	1987.3 ± 21.5	<0.001
Fruits and Vegetables	459.2 ± 4.9	618.2 ± 6.6	<0.001	533.1 ± 6.9	534.2 ± 6.8	489.2 ± 6.9	<0.001
Dairy	*247.5* ± 3.9	223.0 ± 5.3	<0.001	243.6 ± 5.3	244.5 ± 5.2	227.4 ± 5.3	0.037
UPF groups (g/day):							
Cake and cookies	*3.6* ± 0.1	2.1 ± 0.2	<0.001	1.1 ± 0.2	2.5 ± 0.2	6.1 ± 0.2	<0.001
Soft drinks	*59.8* ± 2.3	40.7 ± 3.2	<0.001	21.5 ± 3.1	46.1 ± 3.0	94.5 ± 3.1	<0.001
Sweets	*20.3* ± 0.6	15.8 ± 0.8	<0.001	6.1 ± 0.7	14.3 ± 0.7	37.5 ± 0.7	<0.001
Processed meat	*3.2* ± 0.2	1.5 ± 0.3	<0.001	0.8 ± 0.3	1.6 ± 0.3	5.9 ± 0.3	<0.001
Bread	*327.9* ± 3.5	337.2 ± 4.8	0.135	374.0 ± 4.6	337.7 ± 4.6	282.9 ± 4.6	<0.001
Margarine	*0.3* ± 0.03	0.3 ± 0.04	0.825	0.1 ± 0.04	0.3 ± 0.04	0.4 ± 0.04	<0.001
mayonnaise and dressings	*1.4* ± 0.1	0.9 ± 0.1	<0.001	0.4 ± 0.1	1.0 ± 0.1	2.5 ± 0.1	<0.001

1 Obtained from ANCOVA (all dietary intakes were adjusted for total energy intake, age and sex).

Data are presented as mean ± SE.

UPF: ultra processed food.

Multivariable-adjusted ORs (95% CI) for PCAD across the tertiles of UPFs consumption are provided in Table 3. Crud model showed that those in the highest tertile of the UPFs consumption had higher chance of PCAD in comparison with those in the lowest tertile (OR: 2.14; 95% CI: 1.78–2.57). Adjustment for confounding variables strengthened this relationship (OR: 2.52; 95% CI: 1.97–3.23). In addition, there was a significant upward trend in PCAD risk as tertiles of UPFs consumption increased (*p* trend < 0.001 for all models).

**Table 3:** ORs (95%CI) of premature coronary artery disease across tertiles of ultra-processed foods consumption.

Variables	UPF tertiles (% of Kcal)			*p*-trend^1^
	T1	T2	T3	
Crude model	1	1.09 (0.92, 1.30)	2.14 (1.78, 2.57)	<0.001
Model 1	1	1.11 (0.91, 1.34)	2.60 (2.10, 3.21)	<0.001
Model 2	1	1.14 (0.92, 1.41)	2.84 (2.23, 3.62)	<0.001
Model 3	1	1.09 (0.87, 1.35)	2.53 (1.98, 3.24)	<0.001
Model 4	1	1.09 (0.87, 1.35)	2.53 (1.97, 3.24)	<0.001
Model 5	1	1.09 (0.87, 1.36)	2.52 (1.97, 3.23)	<0.001

1 *p* trend was determined by ANOVA and ANCOVA in the crude and adjusted models for continuous variables and mantel Hansel for categorical variables.

UPF: ultra processed food.

Model 1: adjusted for age (years), sex (male/female), daily energy intake (Kcal/day).

Model 2: additionally, adjusted for smoking (yes/no), level of education (under graduate/graduate/post graduate), different ethnicities and family history of cardiovascular disease (yes/no).

Model 3: additionally, adjusted for Mediterranean dietary index.

Model 4: additionally, adjusted for body mass index (Kg/m^2^).

Model 5: additionally, adjusted for waist circumference (cm).

Multivariable-adjusted ORs (95% CI) for PCAD severity across the tertiles of UPFs consumption are provided in Table 4. Crud model showed that those in the highest tertile of the UPFs consumption had approximately two times higher chance for having severe PCAD than those in the first tertile (OR: 2.34; 95% CI: 1.97–2.74). In the fully adjusted model, we found a stronger relationship between UPFs consumption and PCAD severity (OR: 2.64; 95% CI: 2.16–3.22). In addition, there was a significant upward trend in odds of PCAD severity, as tertiles of UPFs consumption increased (*p* trend < 0.001 for all models).

**Table 4:** Odds ratio (OR) (95%CI) of premature coronary artery disease severity across tertiles of ultra-processed foods consumption.

Variables	ORs (95%CI) of UPF consumption (% of Kcal)			*p*-trend^1^
	T1	T2	T3	
Crude Model	1	1.11 (0.60, 1.30)	2.34 (1.97, 2.74)	<0.001
Model 1	1	1.15 (0.98, 1.36)	2.83 (2.36, 3.35)	<0.001
Model 2	1	1.21 (1.00, 1.45)	3.00 (2.46, 3.67)	<0.001
Model 3	1	1.13 (0.94, 1.36)	2.64 (2.16, 3.22)	<0.001
Model 4	1	1.13 (0.51, 1.36)	2.61 (2.14, 3.22)	<0.001
Model 5	1	1.13 (0.94, 1.36)	2.64 (2.16, 3.22)	<0.001

1 *p* trend was determined by ANOVA and ANCOVA in the crude and adjusted models for continuous variables and mantel Hansel for categorical variables.

UPF: ultra processed food.

Model 1: adjusted for age (years), sex (male/ female), daily energy intake (Kcal/day).

Model 2: additionally, adjusted for smoking (yes/ no), level of education (under graduate/graduate/post graduate), different ethnicities and family history of cardiovascular disease (yes/no).

Model 3: additionally, adjusted for Mediterranean dietary index.

Model 4: additionally, adjusted for body mass index (Kg/m^2^).

Model 5: additionally, adjusted for waist circumference (cm).

Multivariable-adjusted ORs (95% CI) for PCAD severity in different ethnicity across the tertiles of UPFs consumption are provided in Table 5. We found that in the highest tertile of the UPFs consumption, participants of three ethnicities (1**-**Balouch and Arab and Qashqaei, 2-Fars and 3-Kord) had statistically significant higher chance of PCAD than those in the lowest tertile (OR: 2.56; 95% CI: 1.18–5.57, OR: 2.78; 95% CI: 2.08–3.71 and OR: 6.12; 95% CI: 3.19–11.75, respectively).

**Table 5:** ORs (95%CI) of premature coronary artery disease in different ethnicity across tertiles of ultra-processed foods consumption.

Variables	ORs (95%CI) of UPF consumption (% of Kcal)			*p*-trend^1^
	T1	T2	T3	
*Balouch and Arab and Qashqaei 234 (7%)*				
Crude Model	1	1.00 (0.52, 1.92)	1.84 (0.94, 3.59)	0.071
Model 1	1	1.02 (0.49, 2.10)	2.56 (1.18, 5.57)	0.016
*Fars 1821 (54.5%)*				
Crude Model	1	1.21 (0.96, 1.54)	2.29 (1.79, 2.94)	<0.001
Model 1	1	1.16 (0.89, 1.51)	2.78 (2.08, 3.71)	<0.001
*Azari 298 (8.9%)*				
Crude Model	1	0.96 (0.50, 1.84)	1.21 (0.60, 2.42)	0.620
Model 1	1	1.11 (0.55, 2.26)	1.41 (0.66, 3.04)	0.383
*Gilak 233 (7.0%)*				
Crude Model	1	0.81 (0.42, 1.57)	2.70 (1.22, 5.96)	0.021
Model 1	1	0.69 (0.33, 1.41)	1.97 (0.83, 4.69)	0.195
*Kord 368 (11.0%)*				
Crude Model	1	1.23 (0.75, 2.04)	3.84 (2.24, 6.58)	<0.001
Model 1	1	1.33 (0.76, 2.34)	6.12 (3.19, 11.75)	<0.001
*Lor 178 (5.3%)*				
Crude Model	1	1.48 (0.50, 4.38)	1.26 (0.40, 4.02)	0.613
Model 1	1	3.11 (0.83, 11.66)	2.02 (0.55, 7.36)	0.230
*Bakhtiari 209 (6.3%)*				
Crude Model	1	0.84 (0.44, 1.62)	1.95 (0.91, 4.18)	0.132
Model 1	1	0.88 (0.41, 1.88)	1.94 (0.78, 4.79)	0.207

1 *p* trend was determined by ANOVA and ANCOVA in the crude and adjusted models for continuous variables and mantel Hansel for categorical variables.

UPF: ultra processed food.

Model 1: adjusted for age (years), sex (male/ female), daily energy intake (Kcal/day).

## Discussion

In this multi-centric case-control study, a significant direct relationship was obtained between UPFs consumption and the risk of PCAD. This is the first study that examined the relation between UPFs and PCAD in a population with different ethnicities. We found 2.3 times higher risk of PCAD in the 3rd tertile of UPFs consumption. Although the share of UPFs in daily energy intake in this population was substantially lower than Western populations [6.5 vs. 60% in United Kingdom (31) and in the United States (32)], our results were similar with earlier ones in the magnitude. For instance, the Aragon Workers’ Health Study on middle-aged Spanish worker’s demonstrated that approximately 500 g/day of UPFs consumption was associated with a 2-fold higher prevalence of coronary atherosclerosis compared to the consumption of only 100 g/day (16). In addition, a Brazilian study by da Silva et al. (21) declared that higher UPF consumption was related to increased risk of peripheral arterial disease in the whole sample and women, but this association was not seen in men. A cohort study on nearly 155,000 American adults aged 55–74 also revealed that participants in the highest quintile of UPFs consumption had 68% higher risk of heart disease than those in the first quintile (33). A recent meta-analysis on prospective cohort studies also revealed that highest UPF consumption was associated with a 29% increase in the risk of CVD.

Global interest for consuming UPFs is rapidly increasing. UPFs are high-calorie dense products which are usually consumed in large portion sizes. Due to this fact, higher consumption of UPFs is associated with lower intake of fruits, vegetables, olive oil, nuts and legumes which are high in fiber, PUFAs, anti-oxidants and anti-inflammatory properties which all are cardio-protective (34, 35). Similarly, refined sugars which are numerously used in UPFs products, specifically sugar-sweetened beverages, may cause a delay in satiety signal (36). This, in turn, leads to excessive calorie, fats and sugar intake and consequently excess weight, which are strong risk factor for CVD (37). Beslay et al. (38) declared that each 10% increase in UPFs consumption in 110,260 adult participants from the French prospective population-based NutriNet-Santé cohort study, was associated with an 11% and a 10% increase in the risk of overweight and obesity, respectively. In addition an observational study in Iranian adults declared that men with higher UPFs consumption were twice as likely to be overweight compared with men with the lowest intake, but this association was not observed in women (11).

High amount of salt (39), trans fatty acids (TFAs), SFAs (40) and low amount of fiber of UPFs products might be other contributors to CVD in UPFs. Processed meats, cakes and cereals, crackers and pre-prepared foods contain high amount of salt, which are linked to hypertension, endothelial damages and CVD (41). In a large Spanish cohort study, university educated middle aged in the top tertile of UPFs consumption had 21% greater risk of hypertension than those in the bottom tertile (39). In addition, hydrogenated vegetable oils used in the production of UPFs (40) raises LDL-C and Lp(a) lipoprotein, reduces HDL-C, and increases the ratio of total cholesterol to HDL-C, which altogether increase the risk of CVD (42). Beside this, low amount of fiber and food additives alter the composition of gut microbiota, leading to inflammatory disease, weight gain and CVD (43).

Beyond the nutritional composition of UPFs, other harmful components and mechanisms may play a role. Additives, stabilizers and colors used in UPFs are special concerns for CVD. Phosphate, a known UPFs additive, is usually used in order to enhance appearance, and shelf life (15). Inorganic phosphate of UPFs are absorbed by approximately 90% in gastro intestinal tract (44) which causes calcium deposition in blood vessels at a faster rate (15). The production of acrylamide and acrolein, two compounds which produced during heat treated procedures, have been related to CVD risk (45, 46). Plastic packaging of UPFs products is also a risk factor. Bisphenol A, an industrial substance used in plastic packaging, plays a role in CVD process (47).

This study comes with some limitations. Due to the nature of case–control study, establishing a causal relationship between UPFs and PCAD was not possible. In addition, in the case of using FFQ, recall bias and misclassification of individuals across tertiles of the UPFs are likely. This study also presents important strengths. First, this is the first study which examined the relationship between UPFs and PCAD. Second, using a relatively large sample size from different parts of the country with multiple ethnicities increases the external validity of our findings and therefore our results can be generalized to different populations of Iranians. Third, using the NOVA food classification which is a valid tool for nutrition epidemiologic studies.

## Conclusion

In conclusion, we found that higher consumption of UPFs was related to increased risk of PCAD and higher chance for having severe PCAD in different ethnicities of Iranian adults. Although, future cohort studies are warranted to confirm the results of this study, our findings can increase people’s awareness about the risk of UPFs consumption on cardiovascular health and the necessity to reduce UPFs intake.

## Data availability statement

The raw data supporting the conclusions of this article will be made available by the authors, without undue reservation.

## Ethics statement

The studies involving human participants were reviewed and approved by Medical Ethics Committee in Isfahan, Iran. The patients/participants provided their written informed consent to participate in this study.

## Author contributions

ShA and FH contributed to the concept. FaN contributed to data entry, managing, and cleaning. KT, NM, and FSh contributed to data interpreting. ShA, FH, and NM contributed to manuscript drafting. NM and NiS contributed to study design. SM analyzed the data. EZ, SM, FeN, HA, TK, NA, NS, KS, ML, SG, EJ, AS, MD, MC, AA, HH, SN, SS, ShSh, and RM contributed to the data collection. All authors contributed to the article and approved the submitted version.

## Funding

This study has been funded by the Research and Technology Department, Iran Ministry of Health and Medical Education and the Iranian Network of Cardiovascular Research (296055).

## Conflict of interest

The authors declare that the research was conducted in the absence of any commercial or financial relationships that could be construed as a potential conflict of interest.

## Publisher’s note

All claims expressed in this article are solely those of the authors and do not necessarily represent those of their affiliated organizations, or those of the publisher, the editors and the reviewers. Any product that may be evaluated in this article, or claim that may be made by its manufacturer, is not guaranteed or endorsed by the publisher.

## Abbreviations

UPF: ultra processed food
PCAD: premature coronary artery disease
CVD: cardio vascular disease
FFQ: food frequency questioner
FBS: fasting blood sugar
TG: triglyceride
TC: total cholesterol
HDL-c: high-density lipoprotein cholesterol
LDL-c: low-density lipoprotein cholesterol
MUFA: mono unsaturated fatty acids
PUFA: poly unsaturated fatty acids
SFA: saturated fatty acid
TFA: trans fatty acid
SBP: systolic blood pressure
DBP: diastolic blood pressure
